# Mean platelet volume (MPV) as new marker of diabetic macrovascular complications in patients with different glucose homeostasis: Platelets in cardiovascular risk

**DOI:** 10.1186/s12933-024-02177-3

**Published:** 2024-03-02

**Authors:** Velia Cassano, Giuseppe Armentaro, Domenico Iembo, Sofia Miceli, Teresa V. Fiorentino, Elena Succurro, Maria Perticone, Franco Arturi, Marta L. Hribal, Tiziana Montalcini, Francesco Andreozzi, Giorgio Sesti, Arturo Pujia, Angela Sciacqua

**Affiliations:** 1 Department of Medical and Surgical Sciences, University Magna Graecia of Catanzaro, Catanzaro, 88100 Italy; 2 Research Center for the Prevention and Treatment of Metabolic Diseases (CR METDIS), University “Magna Graecia” of Catanzaro, Catanzaro, 88100 Italy; 3 Department of Clinical and Experimental Medicine, University Magna Graecia of Catanzaro, Catanzaro, 88100 Italy; 4 Department of Clinical and Molecular Medicine, Sapienza University of Rome, Rome, 00185 Italy; 5 Campus Universitario “S. Venuta”, Viale Europa – Località Germaneto 8810, Catanzaro, Italy

**Keywords:** Mean platelets volume, Type 2 diabetes, Pre-diabetes, Arterial stiffness, Global longitudinal strain

## Abstract

**Background:**

Platelets play an important role in the development of cardiovascular disease (CVD). Mean platelet volume (MPV) is considered as biological marker of platelets activity and function. The aim of the present study was to evaluate MPV values and its possible correlation with arterial stiffness and subclinical myocardial damage, in normal glucose tolerance patients (NGT), in newly diagnosed type 2 diabetic (T2DM) patients and in individuals with pre-diabetes.

**Methods:**

We enrolled 400 newly diagnosed hypertensive patients. All patients underwent an Oral Glucose Tolerance test (OGTT). Arterial stiffness (AS) was evaluated with the measurement of carotid-femoral pulse wave velocity (PWV), augmentation pressure (AP) and augmentation index (AI). Echocardiographic recordings were performed using an E-95 Pro ultrasound system.

**Results:**

Among groups there was an increase in fasting plasma glucose (FPG) (*p* < 0.0001), fasting plasma insulin (FPI) (*p* < 0.0001), high sensitivity c reactive protein (hs-CRP) levels (*p* < 0.0001) and a decrease in renal function as demonstrated by e-GFR values (*p* < 0.0001). From the NGT group to the T2DM group there was a rise in MPV value (*p* < 0.0001). Moreover, in the evaluation of arterial stiffness and subclinical myocardial damage, MPV showed a positive correlation with these parameters.

**Conclusions:**

In the present study we highlighted that MPV is significantly increased, not only in newly diagnosed T2DM patients, but also in early stage of diabetes, indicating that subjects with pre-diabetes present increased platelets reactivity. Moreover, our results suggest that MPV is associated with increased arterial stiffness and subclinical myocardial damage, indicating MPV as new marker of CV risk.

## Background

The risk of cardiovascular disease (CVD) is higher in patients with type 2 diabetes (T2DM) and microvascular as well as macrovascular complications are associated with hyperglycaemia [1]. Insulin resistance (IR) has a close association with negative cardiovascular (CV) events; triglyceride-glucose index (TyG), a composite indicator obtained by ln [fasting TG (mg/dL) × fasting plasma glucose (mg/dL)/2], is considered a new marker for IR and recently, several studies proved that TyG is associated with CVD, in particular arterial, in patients with T2DM and pre-diabetes [2, 3].

The mechanism at the base of CV complications of T2DM are already present in pre-diabetes, in fact pre-diabetes, as a precursor state of diabetes, is associated with higher burden of cardiac diseases [4, 5]. According to these evidences, pre-diabetic patients exhibit more vascular complications in comparison to normoglycemic patients.

Platelets play an important role in the development of (CVD) [6]; after a vascular injury, activated platelets secret various substance that mediate inflammation, coagulation, thrombosis and atherosclerosis. Among platelets indices, mean platelet volume (MPV) is an estimation of the average size of platelets and is considered as biological marker of platelets activity and function [7]. MPV provides a precise measure of platelets size and is measured using an automated haematological analyser. Recently, data obtained from several studies demonstrated that increased MPV values could be considered as a potential marker of CVD, in fact, higher MPV values were linked with severity of coronary artery occlusion, presence of angina pectoris and myocardial infarction, as well as development of atherosclerosis [8]. In addition, several studies demonstrated that increased MPV values have been detected in patients with metabolic disorders such as T2DM [9]. Platelets of T2DM patients are characterized by dysregulation of several signalling pathway, resulting in rising platelets activity [10]. Thus, platelets are subjected more frequently to sub-threshold stimuli, resulting in accelerated thrombopoiesis of more reactive platelets [11].

In addition, previous studies showed that poor glycaemic control and increased values of MPV may have a crucial role in the development of microvascular and macrovascular complications related to T2DM [9].

However, it is still not completely clear whether higher MPV is present in early stages of T2DM and how it is correlated with CV complications, in particular with arterial stiffness and subclinical myocardial damage.

Based on previous evidence, the aim of the present study was to evaluate MPV values in normal glucose tolerant (NGT) patients, pre-diabetic and newly diagnosed T2DM patients; in addition, we evaluated the possible correlation of MPV with TyG score, arterial stiffness and subclinical myocardial damage.

## Methods

### Study population

The study population includes 400 Caucasian newly diagnosed hypertensive patients (251 men and 149 women, mean age 60.1 ± 11.9) referring to the Catanzaro Metabolic Risk Factors (CATAMERI) Study [12]. Each recruited subject underwent review of his or her medical history, physical estimation, and anthropometrical evaluation with evaluation of height, weight and BMI.

Exclusion criteria were causes of secondary hypertension, clinical evidence of heart failure, diagnosis of anaemia, history of chronic or malignant respiratory disease, malabsorption diseases, endocrinological pathologies, alcohol or drug abuse. The ethics committee authorized the protocol and each study participant gave informed written consent (code protocol number 2012.63). All evaluations were performed in agreement with the principles of the Helsinki Declaration.

### Blood pressure measurement

The evaluation of clinical Blood Pressure (BP) was performed according to current guidelines. Measurements of BP were acquired in the left arm of patients in sitting position using a semi-automatic sphygmomanometer (OMRON, M7 Intelli IT) after 5 min of rest. BP values were the average of three measurements. This evaluation was repeated on three different occasions at least 2 weeks apart. Subjects with a clinic SBP > 140 mmHg and/or DBP > 90 mmHg were defined as hypertensive [13]. Pulse pressure (PP) values were acquired as the difference between systolic and diastolic BP measurements.

### Laboratory determination

All laboratory determinations were executed after an overnight fasting. A 75-g OGTT was carried out with 0, 30, 60, 90, and 120 min sampling for insulin and plasma glucose measurements. Glucose tolerance status was defined on the basis of OGTT using the American Diabetes Association (ADA) criteria: NGT when fasting plasma glucose was < 100 mg/dL, IFG when fasting plasma glucose was 100–125 mg/dL and 2-hours post load < 140 mg/dL, IGT when fasting plasma glucose was < 100 mg/dL and 2-hours post load 140–199 mg/dL, T2DM when fasting plasma glucose was ≥ 126 mg/dL and 2-hours post load ≥ 200 mg/dl [14]. Plasma glucose was estimated by the glucose oxidation method (Beckman Glucose Analyzer II; Beckman Instruments, Milan, Italy), and plasma insulin concentration was detected by a chemiluminescence-based assay (Roche Diagnostics).

Insulin sensitivity was estimated using the Matsuda index (insulin sensitivity index [ISI]), calculated as follows: 10.000/ square root of [fasting glucose (millimoles per liter) X fasting insulin (milliunits per liter)] X [mean glucose/mean insulin during OGTT]. The Matsuda index is strongly related to euglycemic-hyperinsulinemic clamp, which represents the gold standard test for measuring insulin sensitivity [15].

Serum creatinine was evaluated by an automated technique based on Creatinine Jaffè compensated method for plasma and serum (Roche Diagnostics) implemented in an auto-analyzer. Albumin concentration was estimated with Alb2 kit on a Cobas C6000 analyzer (Roche Diagnostics, Milan, Italy). Triglycerides, total, HDL and LDL cholesterol values were determined by enzymatic methods (Roche Diagnostics, Mannheim, Germany). Values of estimated glomerular filtration rate (e-GFR) were determined by using the equation proposed in the chronic kidney disease epidemiology (CKD-EPI) collaboration [16].

MPV was evaluated using an ADVIA 2120i haematology system (Siemens, Munich, Germany). Venus blood sampling was performed by using tubes containing EDTA and the samples were tested within 1-hour of collection.

The TyG index was calculated with the following formula: ln[fasting TG (mg/dL) × fasting plasma glucose (mg/dL)/2] [17].

### Arterial stiffness evaluation

For the evaluation of arterial stiffness, we used high-fidelity applanation tonometry (Millar) and a validated system pressure wave analysis computer software (Sphygmocor™). Automatic, non-invasive recording of supine brachial blood pressure of the dominant arm after a 30-minute rest was used for pressure calibration (Dinamap Com-pact T; Johnson & Johnson Medical Ltd, Newport, UK). Within 10 min, 5 BP measurements were taken and the average of the last 3 was used for calibration. The radial artery of the dominant arm with the wrist slightly hyperextended was utilized to record pressure waves representing the average of individual pressure waves recorded consecutively for 8 s. Only pressure waves with peak and background pressure variation of individual waves < 5% were used. The central pressure wave was automatically derived from the radial pressures. Pressure wave measurements were acquired at the level of the right carotid artery, resulting in a more precise central AI [18, 19]. In addition, central waveforms were analysed to obtain the time at the peak/shoulder of the first (T1) and second (T2) pressure wave component during systole. The height of the outgoing pressure wave (P1) was identified as the pressure at the peak/shoulder of T1 while the height of the reflected pressure wave (P2) as the pressure at the peak/shoulder of T2, either in absolute terms or as a percentage of the ejection duration. The difference between P2 and P1 was used to define the AP, while AI was defined as [AP/PP] × 100. The carotid and femoral pressure waveforms were analysed to define the aortic PWV. From the foot-to-foot time difference between the carotid and femoral waveforms, the carotid-to-femur transit time (ΔT) was calculated. The distance between the surface marks of the sternal notch and femoral artery was used to estimate the path length between the carotid and femoral arteries (L), and the PWV calculated as L/ΔT [20].

### Echocardiographic measurements

Comprehensive 2D and Doppler echocardiography were acquired using a commercially available ultrasound machine (Vivid E95, GE Healthcare, Horten, Norway) and performed according to the American Society of Echocardiography (ASE) guide lines [21]. Echocardiographic readings were made in random order by the investigator, who had no knowledge of patient’s clinical data.

LVM was calculated using the formula proposed by Devereux et al. and corrected for body surface area (BSA), to derive the LVMI [22]. LV end-diastolic and end-systolic volumes and LVEF were calculated using biplane disk-summation algorithm [21], and the indexed by BSA. The values of all parameters were obtained as the average value of three consecutive cardiac cycles.

A 2D speckle tracking analysis was retrospectively performed using vendor-specific 2D speckle tracking software (EchoPAC PC, version 113.0.5, GE Healthcare, Horten, Norway). Manual tracings of the endocardial border during end-systole in three apical views was performed to evaluate GLS.

### Statistical methods

Normally distributed data are reported as mean ± SD. To test the differences among groups, analysis of variance (ANOVA) was performed for clinical and biological data, followed by the Bonferroni post hoc test for multiple comparisons. Chi-squared test was considered for categorical variables. A linear regression analysis was performed, in the whole study population, to compare MPV, PWV and GLS with different covariates. Afterwards, variables achieving statistical significance were inserted in a multiple stepwise multivariate linear regression model to determine the independent predictor of MPV, PWV and GLS. The accuracy of MPV as a predictor of PWV and GLS was evaluated by processing a ROC curve. The AUC described how MPV values were associated with PWV and GLS. Data were considered significant at *p* < 0.05. All comparisons were performed using the statistical package SPSS 20.0 for Windows (SPSS Inc., Chicago, IL, USA).

## Results

### Study population

**Fig. 1:**
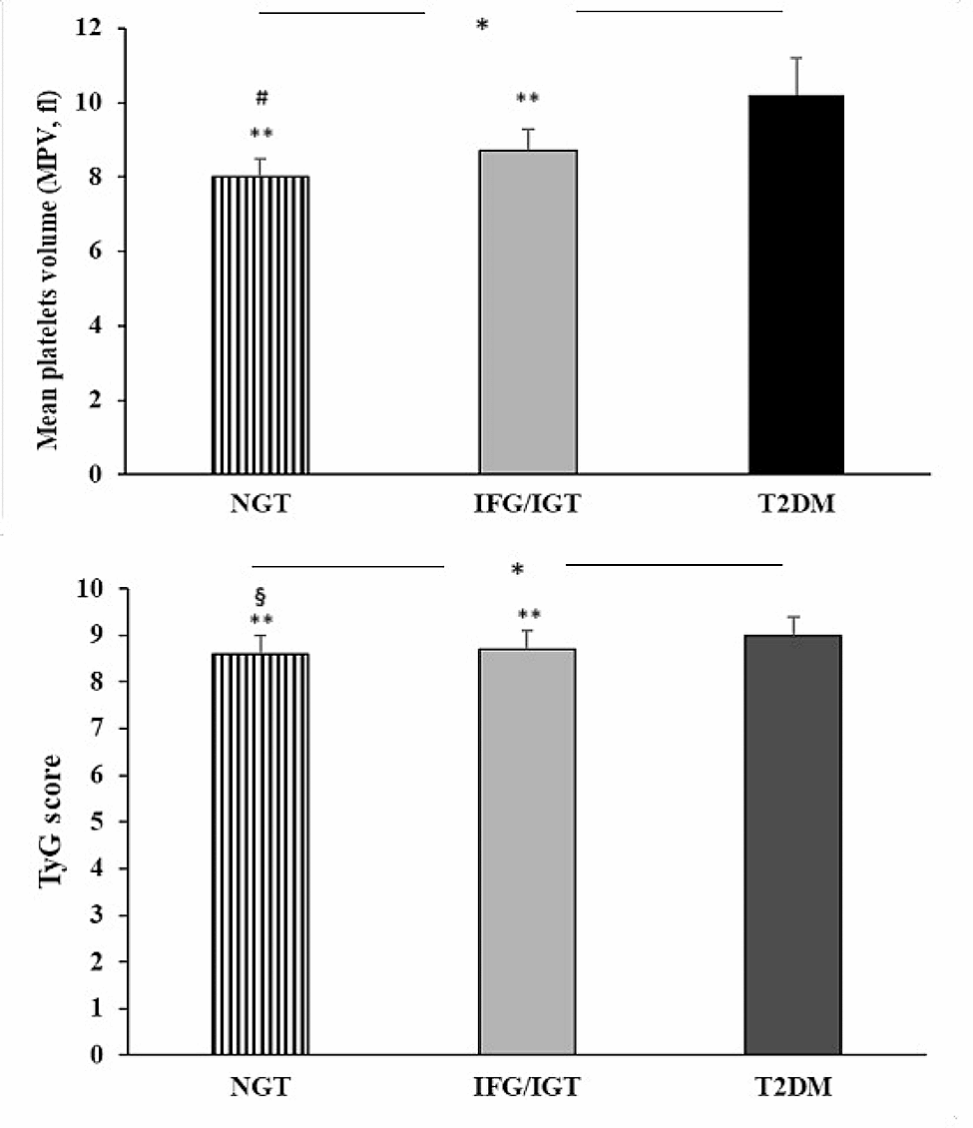
Graphical representation of MPV values and TyG score detected in NGT, IGT/IFG and T2DM patients. Data are mean ± SD. *p < 0.0001 (ANOVA); § *p* = 0.007 IGT/IFG, ** *p* < 0.0001 vs. DM2, # *p* < 0.0001 vs. IFG/IGT (Bonferroni post-hoc test). NGT = Normal Glucose Tolerance; IGT = Impaired Glucose Tolerance; IFG = Impaired fasting glucose T2DM = Type 2 Diabetes Mellitus

The 400 enrolled participants, was divided according to Oral Glucose Tolerance Test (OGTT) values: 179 subjects were NGT, 137 pre-diabetic isolated impaired fasting glucose an impaired glucose tolerance (IFG/IGT) and 84 had newly diagnosed T2DM. Clinical characteristics of subjects are reported in Table 1. At the time of enrolment, patients were not on pre-existing drug therapy. No statistically significant differences were observed, among the three groups, for age, gender, body mass index (BMI), smoking habit, systolic blood pressure (SBP), diastolic blood pressure (DBP), HDL and LDL cholesterol. By contrast,, we observed a significant increase in fasting plasma glucose (FPG) (*p* < 0.0001), 2-hours plasma glucose (*p* < 0.0001), fasting plasma insulin (FPI) (*p* < 0.0001), 2-hours plasma insulin (*p* < 0.0001). In addition, there was a deterioration of insulin sensitivity, as evidenced by Matsuda/index (*p* < 0.0001). Moreover, we observed a significant rise in high sensitivity C reactive protein (hs-CRP) (*p* < 0.0001), HbA1c (*p* < 0.0001), platelets count (PLT) (*p* = 0.003), triglycerides (TG) (*p* = 0.001) values and a reduction in estimated glomerular filtration rate (e-GFR) (*p* < 0.0001). Of interest, from NGT to T2DM group, there was a progressive increase in MPV values (*p* < 0.0001) (Fig. 1); moreover, there was a significant increase in TyG score with the worsening of metabolic status (*p* < 0.0001) (Fig. 1).

**Table 1:** Anthropometric, hemodynamic, and biochemical characteristics of the study population according to glucose tolerance status

Variables	All (*n* = 400)	NGT (*n* = 179)	IFG/IGT (*n* = 137)	T2DM (ND) (*n* = 84)	P^a^
Gender, *m/f* Age, *yrs* BMI, *Kg/m2* SBP, *mmHg* DBP, *mmHg* Smoking habits FPG, *mg/dl* 2-h glucose, *mg/dl* FPI, µ*U/ml* 2-h insulin, µ*U/ml* Matsuda index Hs-CRP, *mg/L* MPV, fl. PLT µ*U/ml* TG, *mg/dl* HDL, *mg/dl* LDL, *mg/dl* HbA1c *mmol/mol* Tyg score e-GFR, *ml/min/1.73m*^*2*^	251/149 60.1 ± 11.9 32.1 ± 10.4 138.0 ± 7.0 78.9 ± 8.2 138/262 98.5 ± 13.3 156.0 ± 50.5 15.4 ± 6.2 111.2 ± 63.3 52.4 ± 30.5 3.3 ± 2.4 8.7 ± 1.1 212.1 ± 65 136.3 ± 61.1 48.2 ± 11.8 109.4 ± 39.0 6.1 ± 0.8 8.7 ± 0.4 96.1 ± 21.5	108/71 59.2 ± 10.7 33.3 ± 14.1 137.3 ± 7.52 78.8 ± 10.2 64/115 89.2 ± 8.1 116.3 ± 19.6 11.9 ± 4.9 79.6 ± 37.2 71.8 ± 35.2 2.7 ± 1.9 8.0 ± 0.5 202.3 ± 53.3 129.0 ± 49.4 49.8 ± 11.3 112.4 ± 38.1 5.9 ± 0.6 8.6 ± 0.4 104.0 ± 20.9	87/50 60.9 ± 12.9 31.8 ± 6.2 138.3 ± 6.4 78 ± 9.4 46/91 102.2 ± 9.3 163.5 ± 29 17.5 ± 5.8 135 ± 68.2 37.9 ± 10.3 3.6 ± 2.9 8.7 ± 0.6 207.7 ± 55.6 133.0 ± 65.5 46.8 ± 11 104.9 ± 38.9 6.2 ± 1.0 8.7 ± 0.4 91.0 ± 21.7	56/28 60.9 ± 12.5 30.3 ± 4.8 139.1 ± 6.5 81.1 ± 9.1 28/56 112.5 ± 12.5 228.4 ± 35.9 19.6 ± 5.4 139.5 ± 69 34.8 ± 12.9 3.8 ± 2.1 10.2 ± 1.0 233.7 ± 59 157.3 ± 71.3 47.1 ± 13.7 111.7 ± 40.5 6.4 ± 0.7 9.0 ± 0.5 87.8 ± 16.6	0.597 0.436 0.08 0.116 0.167 0.928 < 0.0001^*^ < 0.0001^$^ < 0.0001^#^ < 0.0001º < 0.0001^∆^ < 0.0001^€^ < 0.0001^•^ 0.003^@^ 0.001 0.054 0.209 < 0.0001^∑^ < 0.0001^§^ < 0.0001^†^

NGT = Normal Glucose Tolerance; IGT = Impaired Glucose Tolerance; IFG = Impaired fasting glucose T2DM = Type 2 Diabetes Mellitus; BMI = Body Mass Index; SBP = Systolic Blood Pressure; DBP = Diastolic Blood Pressure; FPG = Fasting Plasma Glucose; FPI = Fasting Plasma Insulin; TG = Triglycerides; HDL = high density lipoprotein; LDL = low density lipoprotein; MPV = Mean Platelets Volume; PLT = Platelets; hs-CRP = high-sensitivity C-reactive protein; e-GFR = estimated glomerular filtration rate; HbA1c = Glycated haemoglobin. Data are mean ± SD. ^a^ = Overall difference among groups (ANOVA)

Bonferroni post-hoc analysis:

* NGT vs. IGT/IFG *p* < 0.0001, NGT vs. DM2 *p* < 0.0001, IGT/IFG vs. DM2 *p* < 0.0001

$ NGT vs. IGT/IFG *p* < 0.0001, NGT vs. DM2 *p* < 0.0001, IGT/IFG vs. DM2 *p* < 0.0001

# NGT vs. IGT/IFG *p* < 0.0001, NGT vs. DM2 *p* < 0.0001, IGT/IFG vs. DM2 *p* = 0.016

º NGT vs. IGT/IFG *p* < 0.0001, NGT vs. DM2 *p* < 0.0001

∆ NGT vs. IGT/IFG *p* < 0.0001, NGT vs. DM2 *p* < 0.0001

@ NGT vs. IGT/IFG *p* < 0.0001, NGT vs. DM2 *p* < 0.0001

• NGT vs. IGT/IFG *p* < 0.0001, NGT vs. DM2 *p* < 0.0001, IGT/IFG vs. DM2 *p* < 0.0001

∑ NGT vs. DM2 *p* < 0.0001

† NGT vs. IGT/IFG *p* < 0.0001, NGT vs. DM2 *p* < 0.0001

€ NGT vs. IGT/IFG *p* = 0.016, NGT vs. DM2 *p* < 0.0001

§ NGT vs. IGT/IFG (*p* = 0.007), NGT vs. T2DM (*p* < 0.0001), IGT/IFG vs. T2DM (*p* < 0.0001)

From Bonferroni post-hoc test, T2DM patients exhibited, in comparison to NGT subjects, a worsening of insulin sensitivity as evidenced by Matsuda/index (*p* < 0.0001) and inflammatory profile as shown by hs-CRP (*p* < 0.0001), higher MPV values (*p* < 0.0001), increased PLT count (*p* < 0.0001). Moreover, pre-diabetic and T2DM patients presented higher values of MPV (*p* < 0.0001) when compared to NGT group. In addition, we observed a significant difference, in MPV values, also in the comparison between IGT/IFG group and T2DM group (*p* < 0.0001). Regarding Tyg score, NGT patients exhibited significantly lower values compared to IGT/IFG (*p* = 0.007) and T2DM patients (*p* < 0.0001), moreover prediabetic patients showed lower Tyg score values when compared with T2DM group (*p* < 0.0001).

### Arterial stiffness evaluation and echocardiographic parameters

Peripheral and central hemodynamic parameters are reported in Table 2. We found several differences in arterial stiffness parameters in the three studies groups. As supposed, we observed a significant increase in PWV (*p* < 0.0001), augmentation index (AI%) (*p* < 0.0001), augmentation pressure (AP) (*p* < 0.0001), central-systolic blood pressure (c-SBP) (*p* < 0.0001) and central-pulse pressure (c-PP) (*p* < 0.0001), evidencing an increasing of arterial stiffness with the deterioration of metabolic status. Bonferroni post-hoc test showed that T2DM and IGT/IFG subjects had higher values of AI% (*p* < 0.0001) and AP (*p* < 0.0001), compared to NGT group.

**Table 2:** Arterial stiffness parameters of the study population according to glucose tolerance status

Variables	All (*n* = 400)	NGT (*n* = 179)	IGT/IFG (*n* = 137)	T2DM (ND) (*n* = 84)	P^a^
PWV, *m/s* AI *%* AP, *mmHg* c-SBP, *mmHg* c-DBP, *mmHg* c-PP, *mmHg*	8.1 ± 2.1 20.7 ± 9.4 12.4 ± 3.7 136.1 ± 10.9 80.4 ± 8.7 55.1 ± 11.5	7.7 ± 1.8 16.3 ± 6.2 10.9 ± 2.7 133.5 ± 10.3 79.7 ± 8.8 52.8 ± 12.3	8.0 ± 2.2 24.9 ± 10.1 12.8 ± 4.2 137.3 ± 11.2 80.8 ± 9.2 56.0 ± 11.5	9.2 ± 2.5 23.5 ± 9.7 14.6 ± 3.5 139.7 ± 10.5 81.0 ± 7.8 58.7 ± 8.7	< 0.0001^#^ < 0.0001^§^ < 0.0001^@^ < 0.0001^*^ 0.445 < 0.0001^•^

PWV = pulse wave velocity; AI = augmentation index; AP = augmentation pressure; c-SBP = central systolic blood pressure; c-DBP = central diastolic blood pressure; c-PP = central pulse pressure; NGT = Normal Glucose Tolerance; IGT = Impaired Glucose Tolerance; IFG = Impaired fasting glucose; T2DM = Type 2 Diabetes Mellitus; ^a^ = Overall difference among groups (ANOVA)

Table 3 shows the morphological and functional echocardiographic parameters of the whole population and the three study groups. Proceeding from the first to the third group, there was a significant increase in left ventricular mass (LVM) (*p* < 0.0001), left ventricular mass index (LVMI) (*p* < 0.0001), left ventricular diastolic diameter (LVDD) (*p* < 0.0001), diastolic posterior wall (dPW) (*p* < 0.0001). No statistically significant differences were detected, among the three groups, regarding left global systolic function evaluated as ejection fraction (EF). By contrast, from the first to the third group, there was a progressive decline in left global systolic function evaluated as myocardial deformation and global longitudinal strain (GLS) (*p* < 0.0001). In particular, T2DM and pre-diabetic patients had a more compromised GLS, compared to NGT subjects (*p* < 0.0001).

**Table 3:** Morpho-functional echocardiographic parameters of the study population according to glucose tolerance

Variables	All (*n* = 400)	NGT (*n* = 179)	IGT/IFG (*n* = 137)	T2DM(ND) (*n* = 84)	P^a^
LVDD, *cm* dPW, *cm* LVM, *g* LVMI, *g/m2* LVEF*%* GLS*%* TAPSE, *mm* PAPs, *mmHg* TAPSE/PAPS, *mm/mmHg* E, *m/sec* A, *m/sec* E/A E/e’ LAVI, *ml/mq*	5.1 ± 0.4 0.9 ± 0.1 183.5 ± 33.1 97.7 ± 16.7 60.0 ± 4.1 -19.5 ± 2.7 22.3 ± 2.6 29.4 ± 6.6 0.8 ± 0.2 0.7 ± 0.2 0.8 ± 0.2 0.9 ± 0.3 11.4 ± 2.8 31.2 ± 7.3	4.9 ± 0.4 0.9 ± 0.1 171.0 ± 31.0 91.2 ± 15.9 60.1 ± 4.1 -20.3 ± 2.7 23.0 ± 2.5 28.3 ± 6.9 0.9 ± 0.2 0.8 ± 0.2 0.8 ± 0.2 1.0 ± 0.3 10.6 ± 2.6 29.8 ± 7.1	5.1 ± 0.3 1.0 ± 0.1 188.0 ± 29.1 99.5 ± 13.8 60.0 ± 4.0 -19.1 ± 2.6 22.2 ± 2.6 29.9 ± 6.3 0.8 ± 0.2 0.7 ± 0.2 0.8 ± 0.2 0.9 ± 0.4 11.7 ± 2.6 31.7 ± 7.6	5.2 ± 0.4 1.0 ± 0.1 202.8 ± 33.1 108.4 ± 16.4 59.7 ± 4.1 -18.2 ± 2.5 21.1 ± 2.6 30.9 ± 7.0 0.7 ± 0.3 0.7 ± 0.1 0.9 ± 0.2 0.8 ± 0.2 12.8 ± 2.9 34.7 ± 6.3	< 0.0001^∑^ < 0.0001 < 0.0001^§^ < 0.0001^@^ 0.795 < 0.0001^#^ < 0.0001 0.006 < 0.0001 < 0.0001 0.066 < 0.0001 < 0.0001 0.001^Ω^

LVDD = Left Ventricular diastolic diameter; dPW = diastolic posterior wall; LVM = Left Ventricular Mass; LVMI = Left ventricular Mass Index; LVEF = Left Ventricular Ejection Fraction; GLS = Global longitudinal strain; TAPSE = Tricuspid annular plane systolic excursion; s-PAP = systolic pulmonary arterial pressure; LAVI = left atrial volume index; E = Wave E; A = Wave A. Data are mean ± SD. ^a^ = Overall difference among groups (ANOVA)

Bonferroni post-hoc analysis:

@ NGT vs. IGT/IFG *p* < 0.0001, NGT vs. DM2 *p* < 0.0001, IGT/IFG vs. DM2 *p* < 0.0001

# NGT vs. IGT/IFG *p* < 0.0001, NGT vs. DM2 *p* < 0.0001, IGT/IFG vs. DM2 *p* = 0.043

Ω NGT vs. DM2 *p* < 0.0001

∑ NGT vs. IGT/IFG *p* < 0.0001

§ NGT vs. IGT/IFG *p* = 0.001, NGT vs. DM2 *p* = 0.001, IGT/IFG vs. DM2 *p* = 0.002

Regarding the indices of right global systolic function, we observed a significant reduction in TAPSE values (*p* < 0.0001) with the worsening of metabolic status. As for diastolic function, the E/A ratio was significantly lower in T2DM group (*p* < 0.0001).

Relative to the left atrial volume index (LAVI) parameter, there was a progressive increase from healthy subjects to T2DM patients (*p* = 0.001).

### Linear regression analysis

A linear regression analysis was performed between MPV, GLS and PWV considered as dependent variables and different covariates (Table 4).

**Table 4:** Linear regression analysis between the dependent variables MPV (A), GLS, PWV and different covariates in the entire study population

	MPV	GLS	PWV	
	R/P	R/P	R/P	
Gender, m/f	0.077/0.065	0.012/0.407	-0.012/0.407	
Age, yrs	0.107/0.018	0.009/0.429	0.053/0.151	
BMI, *Kg/m2*	0.165/0.001	-0.059/0.122	0.061/0.114	
HbA1c, *mmol/mol*	0.251/<0.0001	--	--	
Hs-CRP, *mg/L*	0.120/0.009	0.100/0.038	0.111/0.015	
Matsuda index	-0.446/<0.0001	-0.299/<0.0001	-0.382/<0.0001	
TyG score	0.273/<0.0001	0.177/<0.0001	0.111/0.026	
e-GFR, *ml/min/1.73m*^*2*^	0.399/<0.0001	-0.112/0.014	-0.152/0.001	
PLT, µ*U/ml*	0.187/<0.0001	--	--	
MPV, fl.	--	0.383/<0.0001	0.451/<0.0001	
SBP, *mmHg*	--	0.159/0.001	0.071/0.082	
DBP, *mmHg*	--	0.126/0.006	-0.043/0.200	
E/e’	--	0.409/<0.0001	--	
LVMI, *g/m2* HDL, *mg/dl* LDL, *mg/dl*	-- -- --	0.329/<0.0001 -- --	-- -0.140/0.003 0.109/0.016	

BMI = Body Mass Index; **e-GFR =** estimated glomerular filtration rate; hs-CRP = high-sensitivity C-reactive protein; PLT = Platelets; HbA1c = Glycated haemoglobin; MPV = Mean Platelets Volume; SBP = Systolic Blood Pressure; DBP = Diastolic Blood Pressure; LVMI = Left ventricular Mass Index; GLS = Global Longitudinal Strain; TG = Triglycerides; HDL = high density lipoprotein; LDL = low density lipoprotein; PWV = Pulse Wave Velocity

MPV was significantly and directly correlated with HbA1c (*r* = 0.251, *p* < 0.0001), hs-CRP (*r* = 0.120, *p* = 0.009), PLT (*r* = 0.187, *p* < 0.0001), TyG score (*p* = 0.273, *p* < 0.0001), BMI (*r*= -0.165, *p* = 0.001) and age (*r* = 0.107, *p* = 0.018) and inversely correlated with Matsuda index (*r*= -0.446, *p* = < 0.0001) and e-GFR (*r*= -0.399, *p* < 0.0001).

Subsequently, GLS was significantly and directly correlated with E/e’ (*r* = 0.409, *p* = < 0.0001), LVMI (*r* = 0.329, *p* = < 0.0001), MPV (*r* = 0.383, *p* < 0.0001), Hs-CRP (*r* = 0.100, *p* = 0.038), SBP (*r* = 0.159, *p* = 0.001), DBP (*r* = 0.126, *p* = 0.006) and TyG score (*r* = 0.177, *p* < 0.0001) and inversely correlated with Matsuda index (*r*= -0.299, *p* < 0.0001) and e-GFR (*r*= -0.112, *p* = 0.014).

When PWV was considered a dependent variable, it resulted significantly and directly correlated with MPV (*r* = 0.451, *p* < 0.0001), hs-CRP (*r* = 0.111, *p* = 0.015), LDL cholesterol (*r* = 0.109, *p* = 0.016) and TyG score (*r* = 0.111, *p* = 0.026) and inversely correlated with Matsuda index (*r*= -0.382, *p* < 0.0001), e-GFR (*r*= -0.152, *p* = 0.001) and HDL cholesterol (*r*= -0.140, *p* = 0.003).

Variables reaching statistical significance were introduced in a stepwise multivariate linear regression model to identify the independent predictors of MPV, PWV and GLS (Table 5).

**Table 5:** Stepwise multiple regression analysis between MPV, GLS, PWV and different covariates in the entire study population

MPV			
	R2partial	R2 total	*P*
Matsuda/Index e-GFR, *ml/min/1.73m*^*2*^ HbA1c, *mmol/mol* PLT, µ*U/ml*	19.7% 7.7% 1.7% 1.1%	19.7% 27.4% 29.1% 30.2%	< 0.0001 < 0.0001 0.002 0.007
GLS			
	R2partial	R2 total	*P*
E/e’ MPV, fl. LVMI, *g/m2*	16.5% 8.3% 2.0%	16.5% 24.8% 26.8%	< 0.0001 < 0.0001 0.001
PWV			
	R2partial	R2 total	*P*
MPV, fl. Matsuda/Index, *mg/dl* LDL, *mg/dl*	20.1% 3.5% 1.2%	20.1% 23.6% 24.8%	< 0.0001
			< 0.0001 0.032

e-GFR = estimated glomerular filtration rate; MPV = Mean Platelets Volume; PLT = Platelets; HbA1c = Glycated haemoglobin; HbA1c = Glycated haemoglobin; LVMI = Left ventricular Mass Index; LDL = low density lipoprotein; GLS = Global Longitudinal Strain; PWV = Pulse Wave Velocity

In the whole study population, Matsuda index resulted the major predictor of MPV, accounting for 19.7% of its variation; e-GFR, HbA1c and PLT added respectively another 7.7%, 1.7% e 1.1%. Moreover, E/è was the main predictor of GLS justifying 16.5% of its variation; MPV was the second predictor explaining 8.3% of its variation, LVMI added another 2.0%. Considering PWV, MPV was the main predictor explaining 20.1% of its variation, Matsuda index and LDL added respectively 3.5% and 1.2%.

### ROC curve analysis

**Fig. 2:**
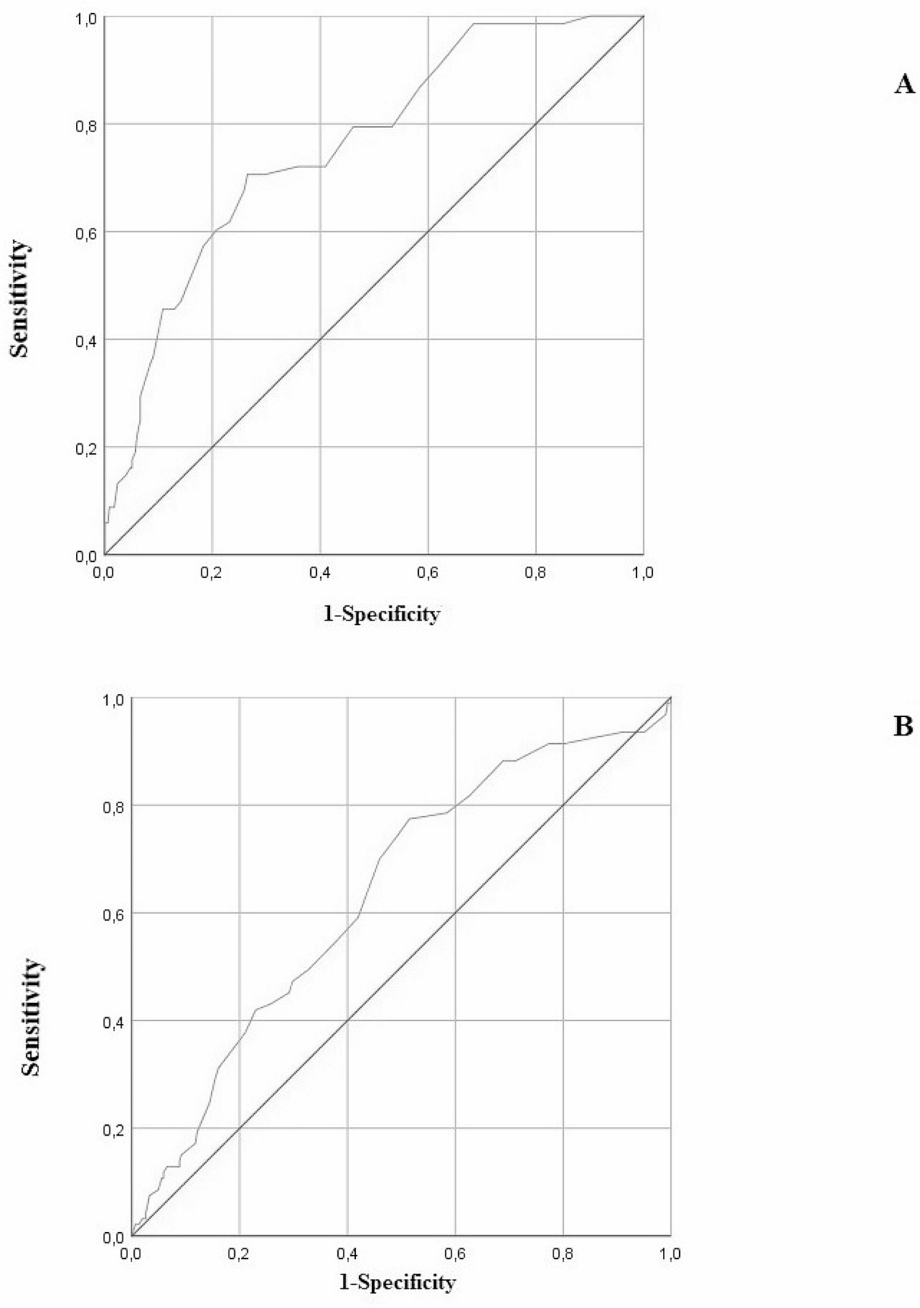
Analysis of ROC curves for the identification of MPV stratified according PWV (**A**) and GLS (**B**)

To further clarify the diagnostic value of MPV for arterial stiffness and subclinical myocardial damage, we performed the receiver operating characteristic (ROC) curve analysis. We used PWV as the gold standard of arterial stiffness; the considered cut-off was 10 m/s. The GLS was the gold standard for judging subclinical myocardial damage severity; the considered cut-off was − 17%. ROC curve analysis showed that the area under curve (AUC) values of MPV for PWV and GLS were respectively 0.76 (standard error 0.030, 95% CI 0.70–0.82, *p* < 0.0001) and 0.64 (standard error 0.033, 95% CI 0.57–0.70, *p* < 0.0001) (Fig. 2).

## Discussion

Of particular interest, the present study highlighted a significant increase in MPV values in the early stage of diabetes according to the results in the IFG/IGT group and a correlation with arterial stiffness and subclinical myocardial damage. In this study, we examined the association between MPV and diabetic disease, in fact, linear regression analysis showed a significant correlation between MPV, HbA1c and Matsuda/index, a known index for insulin sensitivity. Moreover, in the present study we showed that MPV is correlated also with TyG score, a new marker of IR, stressing the concept that platelets activation is linked metabolic disorders. Our obtained data are in agreement with a previous study conducted in a Japanese population, stratified by different glucose tolerance, that demonstrated an increase in MPV values in the early stages of T2DM [7]. However, the mechanisms underlying the increase in MPV in pre-diabetic and T2DM individuals remain not fully explained. One of the possible mechanisms is that insulin resistance is a common denominator of several metabolic conditions related to platelet over activity [23]. Therefore, insulin resistance may cause the lack of the physiological action exerted by insulin on platelet function, such as the reduction of pro-aggregating properties of agonists, and activation of nitric oxide (NO) synthase, with increased NO formation and intraplatelet concentrations of cyclic adenosine monophosphate (cAMP) [23]. Moreover, hyperglycemia increases thrombopoietin production by neutrophils in the liver through the receptor for advanced glycation end products; these processes can lead to an increase in platelet size [7].

Furthermore, we examined MPV as a biomarker correlated to arterial stiffness and subclinical myocardial damage in patients with different glucose tolerance. The present study demonstrated a significant association between increased MPV and progression of arterial stiffness in pre-diabetic and diabetic patients. Of particular interest, linear regression analysis showed that increased PWV, the gold standard of arterial stiffness, was significantly correlated with increased MPV, moreover from stepwise multivariate linear regression model, MPV resulted the major predictor of PWV variation. The analysis of the processed ROC curve and the measurement of the relative AUC also demonstrated the accuracy of MPV as a predictor arterial stiffness. These data suggest an existing link between platelets hyper-activation and atherosclerosis in patients pre-diabetes, highlighting the high CV risk present in this phenotype of patients. High MPV values are considered a potential marker of platelet activation and increased arterial vessel wall stiffness [24].

MPV had already been examined as an index of platelet function and associated to other markers of platelet’s activation. As it is well known, larger platelets are enzymatically and metabolically more active, secrete more serotonin and produce more thromboxane A2, than smaller platelets [25]. All these factors may lead to vascular complication. In fact, activated platelets release numerous substances that control vascular permeability, regulate vasoconstriction or vasodilation, stimulate macrophage recruitment and infiltration, release reactive oxygen species (ROS), and stimulate ROS generation within the vascular wall resulting in decreased vascular NO bioavailability. In addition, activated platelets liberate more platelet-derived growth factors that stimulate the proliferation of smooth muscle cells [26]. Moreover, the over activity of matrix metalloproteinase (MMPs), due to hyper activated platelets leads to a reduction in arterial elasticity by degradation of elastic fibres [26]. Moreover, it is known that chronic inflammation is also linked to arterial stiffness and T2DM [27]; as we can see from our obtained data, hs-CRP resulted positively correlated with MPV and PWV, underlying the exiting link between inflammation, arterial stiffness and platelets activity. In fact, it would seem that circulating platelets size is dependent from the levels of systemic inflammation.

For what concern subclinical myocardial damage, data showed that myocardial deformation indices are relevant in patients with IGT/IFG and T2DM, compared to NGT patients. In particular, the GLS, the gold standard parameter for assessment of myocardial deformation, was found to be significantly increased in relation to increased values of MPV. Moreover, stepwise multivariate linear regression showed that the second predictor of GLS was MPV, pointing to it as a useful parameter for detecting subclinical myocardial damage.

A previous study demonstrated that patients with ischemic or idiopathic cardiomyopathy presented elevated MPV values and functional impairment of the left ventricle associated with increased MPV values [28].

Blood stasis, caused by a dilated chamber and poorly contracted ventricle could be a facilitating or triggering factor, leading to platelet stimulation and ultimately increased MPV [29].

Recently, a meta-analysis evidenced that patients with coronary artery disease (CAD) manifested increased MPV values compared to healthy subjects; moreover, the probability of CAD was higher in patients with high MPV values than in patients with lower MPV [30]. Data from meta-analysis supported the possible role of MPV as a prognostic marker of future CV events, myocardial infarction, and death in patients with CAD.

## Conclusion

In conclusion, data obtained from the present study highlighted an increase in MPV values in pre-diabetic and T2DM patients; in addition, the novelty of our study is that the obtained data suggest that increased MPV is correlated with increased arterial stiffness and progression of myocardial deformation already at an early stage of T2DM, in particular GLS has a prognostic value for CV events in patients with no history of CV complications. Therefore, measurement of MPV could be a useful indicator for determining pre-diabetic and T2DM induced macrovascular complications. Consequently, it would be interesting to establish the cut-off value of MPV for the presence of CV disease, increased risk of disease development, increased risk of thrombotic complications, and increased risk of death. However, this aspect of MPV assessment, which would be required in order to extend its use to the clinical practice, is limited and requires further study.

## Data Availability

No datasets were generated or analysed during the current study.
